# Efficacy of Immune Checkpoint Inhibitors in Rare Tumours: A Systematic Review

**DOI:** 10.3389/fimmu.2021.720748

**Published:** 2021-09-20

**Authors:** Fausto Petrelli, Francesca Consoli, Antonio Ghidini, Gianluca Perego, Andrea Luciani, Paola Mercurio, Alfredo Berruti, Salvatore Grisanti

**Affiliations:** ^1^Oncology Unit, Azienda Socio Sanitaria Territoriale (ASST) Bergamo Ovest, Treviglio, Italy; ^2^Medical Oncology Unit, Department of Medical and Surgical Specialties, Radiological Sciences and Public Health, University of Brescia, Azienda Socio Sanitaria Territoriale (ASST) Spedali Civili of Brescia, Brescia, Italy; ^3^Oncology Unit, Casa di cura Igea, Milano, Italy; ^4^Pharmacy Unit, San Raffaele Hospital, Milano, Italy; ^5^Pathology Unit, Azienda Socio Sanitaria Territoriale (ASST) Bergamo Ovest, Treviglio, Italy

**Keywords:** immunotherapy, rare tumours, systematic review, survival, anti-PD-(L)1 agents

## Abstract

**Background:**

Rare cancers, as defined by the European Union, occur in fewer than 15 out of 100,000 people each year. The International Rare Cancer Consortium defines rare cancer incidence as less than six per 100,000 per year. There is a growing number of reports of the efficacy of immune checkpoint inhibitor (ICI) therapy in patients with rare tumours, and hence, we conducted a comprehensive review to summarise and analyse the available literature.

**Methods:**

A literature search of PubMed was performed on January 31, 2021, using the following ICI names as keywords: ipilimumab, tremelimumab, cemiplimab, nivolumab, pembrolizumab, avelumab, atezolizumab, and durvalumab. Studies on patients with rare tumours who were being treated with ICIs were included. We plotted the overall response rate against the corresponding median survival across a variety of cancer types using linear regression.

**Results:**

From 1,255 publications retrieved during the primary search, 62 publications were selected (with a total of 4,620 patients). Only four were randomised trials. A minority were first-line studies, while the remaining were studies in which ICIs were delivered as salvage therapy in pretreated patients. There was a good correlation between response rate and overall survival (Spearman R^2^ >0.9) in skin cancers, mesothelioma, and sarcomas.

**Conclusions:**

Treatment of advanced-stage rare tumours with ICI therapy was found to be associated with significant activity in some orphan diseases (e.g., Merkel cell carcinoma) and hepatocellular carcinoma. Several ongoing prospective clinical trials will expand the knowledge on the safety and efficacy of ICI therapy in patients with these rare cancers.

## Introduction

In the European Union (EU), rare cancers are defined as those with an incidence of less than six per 100,000 people per year. The Surveillance of Rare Cancers in Europe (RARECARE) project calculated that around four million people in the EU are affected by rare cancers and estimated the annual incidence rate of all rare cancers in Europe as about 108 per 100,000 people, corresponding to one-quarter of all malignancy diagnoses. A list of RARECARE cancers has been identified based on the above epidemiological criterion (1). The Information Network on Rare Cancers (RARECAREnet) project integrates any updated epidemiological information about rare cancers in the EU and provides indicators at the country level and time trends, studying to what extent treatment is centralised in Europe.

Rare cancers are a heterogeneous group of almost 200 cancers with a 5-year survival rate lower than that of more common cancers (49% versus 63%) (2). Beyond the influence of classical prognostic factors such as age, stage, or performance status, the prognosis of patients with rare malignancies is affected by additional factors. These include a lack of medical expertise or insufficient evidence-based guidelines in managing these diseases and difficulties in conducting clinical trials with sufficient statistical power due to the low number of patients affected (3). Furthermore, rare cancers often display intrinsic biological characteristics that may differ from their “common” counterparts and are generally poorly studied. Therefore, rare cancers are also neglected in terms of pharmaceutical research, which translates into fewer therapeutic options for affected patients.

In the past few years, immune checkpoint inhibitors (ICIs) have revolutionised the therapeutic approach to different haematological and solid malignancies, and their efficacy has recently been explored in the rare cancer setting (4). However, the extent of the actual clinical benefit and the strength of the cumulative evidence are largely elusive or contradictory. This could result in misleading conclusions that immunotherapy is futile for some rare cancers and ultimately hamper the future development of immunotherapy trials and the identification of predictive factors in this setting.

Herein, we conducted and reported on a systematic review of the literature on the role of ICIs in patients with rare solid cancers.

## Materials and Methods

This systematic review was performed following the 2020 Preferred Reporting Items for Systematic Reviews and Meta-analyses (PRISMA) reporting guideline. We performed a literature search using PubMed and Embase on January 31, 2021, with the following keywords to identify all studies that reported the efficacy of ICIs in patients affected by rare solid tumours: phase 2, phase 3, ipilimumab, tremelimumab, cemiplimab, nivolumab, pembrolizumab, avelumab, atezolizumab, and durvalumab. Four investigators from two different institutions (FP, AG, FC, and SG) independently screened published articles and meeting abstracts. The inclusion criterion was that the studies described patients with rare solid tumours treated with ICI therapy for advanced stage cancer. The exclusion criteria were haematological malignancies, phase 1 studies, conference abstracts, and a lack of evaluation of ICI therapy. Data extracted by the four authors were the type of disease, the number of patients, treatment, line of therapy, median follow-up, overall response rate (ORR), median progression-free survival (PFS), and overall survival (OS). For inclusion, rare cancers had to belong to the classification reported by RARECAREnet (http://rarecarenet.istitutotumori.mi.it/fact_sheets.php, last accessed December 12, 2020). OS was plotted against ORR, and a linear regression model was fitted. A Spearman correlation coefficient R^2^ value of 0.70 or greater was considered a strong correlation, and an R^2^ value between 0.50 and 0.70 was considered a moderate correlation.

We used descriptive statistics to summarise the study findings. Statistical calculations were performed using Microsoft Excel.

## Results

**Figure 1:**
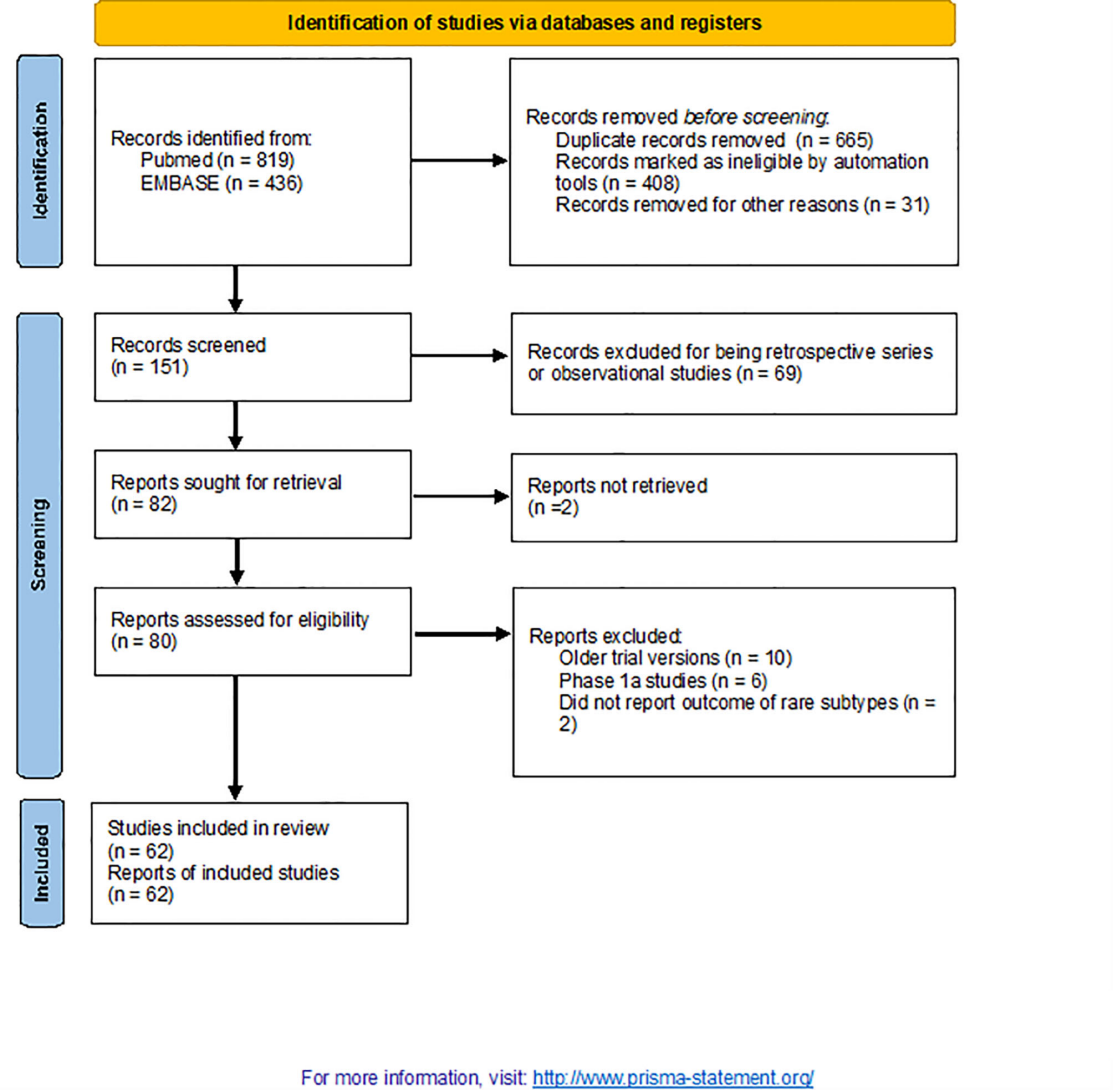
Flow diagram of included studies.

From 1,255 publications retrieved during a primary search, 62 were selected for this study (including 4,620 patients; **Figure 1**) (1, 4–64). Patient characteristics are listed in **Table 1**. Papers were published between 2013 and 2021. The included studies focussed on the following conditions: skin cancers [n = 14; non-cutaneous melanoma, cutaneous squamous cell carcinoma (SCC) and Merkel cell carcinomas (MCC)], rare thoracic tumours (n = 13; mainly mesothelioma), endocrine malignancies (n = 11), hepatobiliary cancers (n = 10), sarcomas (n = 9), testicular cancers (n = 3), and salivary gland tumours (n = 2). All were phase 2 (single-arm) studies, except six that were randomised trials (n = 3 phase 2 and n = 3 phase 3) and two that were phase 1b or both phases 1b and 2 studies. One publication was a pooled analysis of various studies that included nivolumab. Most of the trial arms included single agents (n = 41); doublets of ICIs or ICIs + other agents were included in 21 trials. Only a minority were first-line studies, while the remaining were studies in which ICIs were delivered as salvage therapy in pretreated patients. None were biomarker-driven studies.

**Table 1:** Characteristics of included studies.

Author/year	Type of tumor	Type of study/median follow up (months)	Treatment	Line of therapy	No. of patients	ORR (%)	Median PFS (months; 95%CI)	Median OS (months; 95%CI)	Main AEs (>5%)
**Highly responsive tumours (response rate >20%; median PFS >6 months; median OS > 12–24 months)**									
**skin cancers and non-cutaneous melanoma**									
**D’Angelo/2018** (54)	Merkel cell carcinoma	Phase 2/5.1	AVE	1st	39	62.1	9.1 (–)	–	NR
**D’Angelo/2017** (52)	Mucosal melanoma	Pooled analysis/157	NIVO/NIVO + IPI/IPI	Various	157	23.3 *vs*. 37.1 *vs*. 8.3	3 (2.2–5.4) *vs*. 5.9 (2.8–nr) *vs*. 2.7 (2.6–2.8)	–	Fatigue, diarrhoea, rash
**Johnson/2019** (15)	Uveal melanoma	Phase 2/11.1	PEMBRO	Advanced	5	20	11.0 (–)	nr	Rare (NR)
**Joshua/2015** (16)	Uveal melanoma	Phase 2/11	TREME	Advanced	11	0	2.9 (2.8–3.0)	12.8 (3.8–19.7)	Nausea, diarrhoea, pain
**Kaufman/2018** (19)	Merkel cell carcinoma	Phase 2/16.4	AVE	Pretreated	88	33	2.7 (1.4–6.9)	12.9 (7.5–nr)	NR
**Maubec/2020** (26)	SCC	Phase 2/22.4	PEMBRO	1st	39	41	6.7 (–)	25.3 (14.2–ne)	Fatigue, diarrhoea, hypothyrodism
**Migden/2018** (30)	SCC	Phase 2/7.9	CEMI	Advanced	59	47	nr	nr	Diarrhoea, fatigue, constipation
**Migden/2020** (29)	SCC	Phase 2/9.3	CEMI	Advanced	78	44	nr	nr	Fatigue, diarrhoea, pruritus
**Naing/2020** (5)	SCC	Phase 2/–	PEMBRO	Pretreated	19	31	–	–	Fatigue, rash, hypothyroidism
**Nathan/2019** (62)	Mucosal, acral and uveal melanoma	Phase 2	NIVO	Pretreated	221	–	–	11.5 (6.4–15.0; mucosal) 25.8 (15.1–30.6; acral) 12.6 (10.2–15.1; uveal)	Rash, hypothyroidism, diarrhoea
**Nghiem/2019** (32)	Merkel cell carcinoma	Phase 2/14.9	PEMBRO	1st	50	56	16.8 (4.6–ne)	Nr	Hypothyroidism, pneumonitis
**Nomura/2020** (33)	Mucosal melanoma	Phase 2/18	NIVO	Advanced	20	23.5	1.4 (1.2–2.8)	12.0 (3.5–nr)	Pruritus, rash
**Schadendorf/2019** (61)	Mucosal, acral and uveal melanoma	Phase 2/14.3	NIVO	Pretreated	221	–	–	11.5 (6.4–15.0; mucosal) 25.8 (15.1–30.6; acral) 12.6 (10.2–15.1; uveal)	Skin endocrine and gastrointestinal
**Zimmer/2015** (50)	Uveal melanoma	Phase 2/–	IPI	Advanced	34	0	2.8 (2.5–2.9)	6.8 (3.7–8.1)	Diarrhoea, AST, ALT ↑
***Gastrointestinal cancers***									
**El-Khoueiry/2017** (65)	HCC	Phase2/–	NIVO	1st–2nd (cohorts 1,2)	113^	23 & 22	5.4 (3.9–8.5) & 4.0 (2.6–6.7)	nr & 13.2 (8.6–nr)	Rash, AST increase, pruritus
**Feng/2020** (9)	Biliary	Phase 2/12.8	CDDP + GEM + NIVO	1st–2nd	32	55.6	6.1 (3.4–8.2)	8.5 (5.0–12.5)	Nausea, neutropenia, fatigue
**Feun/2019** (10)	HCC	Phase 2/17	PEMBRO	1st–2nd	29	32	4.5 (2.0–7.0)	13.0 (7.0–nr)	Rash, fatigue, ALT and bilirubin ↑
**Finn/2020** (1)	HCC	Phase 3/13.8	PEMBRO *vs*. BSC	Pretreated	413	18.3 *vs*. 4.4	3.0 (2.8–4.1) *vs*. 2.8 (2.5–4.1)	13.9 (11.6–16) *vs*. 10.6 (8.3–13.5)	Fatigue, AST and bilirubin ↑
**Finn/2020** (11)	HCC	Phase 3/8.6	ATEZO + BEV *vs*. sorafenib	1st	501	27.3 *vs*. 11.9	6.8 (5.7–8.3) *vs*. 4.3 (4.0–5.6)	nr *vs*. 13.2 (10.4–nr)	Hyperthension, fatigue and proteinuria
**Boileve/2020** (17)	Biliary	Phase 2/9.8	DURVA + TREME ± paclitaxel	Pretreated	20	5	–	–	Colitis, fever, abdominal pain
**Kim/2020** (20)	Biliary	Phase 2/12.4	NIVO	Pretreated (2nd–3rd)	54	11°	3.6 (2.3-5.6)	nr	Alkaline phosphatase ↑, lymphopenia, AST ↑, fatigue
**Klein/2020** (22)	Biliary	Phase 2/–	NIVO + IPI → NIVO	Pretreated (85%)	39	23	2.9 (2.2–4.6)	5.7 (2.7–11.9)	NR
**Sangro/2013** (42)	HCC	Phase 2/–	TREME	Pretreated	17	17.6	6.4 (3.9–9.1) TTP	8.2 (4.6–21.3)	Rash, fatigue, anorexia
**Zhu/2018** (49)	HCC	Phase 2/12.3	PEMBRO	2nd	104	17	4.9 (3.4–7.2)	12.9 (9.7–15.5)	Fatigue, pruritus, diarrhoea
***Thoracic cancers***									
**Baas/2021** (63)	Mesothelioma (pleural)	Phase 3/29.7	NIVO + IPI *vs*. CT	1st line	713	40 *vs*. 43	6.8 (5.6–7.4) *vs*. 7.2 (6.9–8)	18.1 (16.8–21.4) *vs*. 14.1 (12.4–16.2)	NR
**Calabrò/2015** (28)	Mesothelioma (pleural)	Phase 2/21.3	TREME	Pretreated	29	3.4	6.2 (5.7–6.7)	11.3 (3.4–19.2)	NR
**Calabrò/2018** (38)	Mesothelioma (pleural)	Phase 2/19.2	TREME + DURVA	1st–2nd line	40	25	5.7 (1.7–9.7)	16.6 (13.1–20.1)	Skin, gastrointestinal
**Cho/2018** (51)	Thymic carcinoma/thymoma	Phase 2/14.9	PEMBRO	Pretreated	33	21	6.1 (5.3-6.9)	14.9 (–)*	Hepatitis, myocarditisi, myasthenia gravis
**Disselhorst/2019** (8)	Mesothelioma (pleural)	Phase 2/14.3	NIVO + IPI	Pretreated	36	29	6.2 (4.1–nr)	nr	Infusion reactions, fatigue, skin disorders
**Giaccone/2018** (12)	Thymic carcinoma	Phase 2/20	PEMBRO	Pretreated	40	22.5	4.2 (2.9–10.3)	24.9 (15.5–nr)	Fatigue, AST and ALT ↑
**Katsuya/2019** (18)	Thymic carcinoma	Phase 2/14.1	NIVO	Pretreated	15	0	3.8 (1.9–7.0)	14.1 (11.1–nr)	Hypoalbuminemia, anemia
**Kim/2020**	NSCLC (sarcomatoid)	Phase 2/12	DURVA + TREME	Pretreated (61%)	18	26.7	5.9 (1.9–11.9)	15.4 (11.1–nr)	Rash, pruritus, pneumonitis
**Maio/2018** (24)	Mesothelioma (pleural 95%)	Phase 2b/–	TREME	2nd–3rd	382	4.5	–	7.7 (6.8-8.9)	Diarrhoea, dyspnea, anorexia
**Nowak/2020** (34)	Mesothelioma (pleural)	Phase 2/28.2	CDDP + PEME + DURVA	1st	54	48	7.0 (5.7–9.0)	18.4 (13.1–24.8)	Constipation, fatigue, nausea
**Okada/2019** (35)	Mesothelioma (pleural)	Phase 2/16.8	NIVO	2nd–3rd	34	29	6.1 (2.9–9.9)	17.3 (11.5–nr)	Infection, weight increase
**Quispel–Janssen/2018** (37)	Mesothelioma (pleural)	Phase 2/27.5	NIVO	Pretreated	34	24	2.6 (2.2–5.4)	11.8 (9.7–15.7)	NR
**Scherpereel/2019** (43)	Mesothelioma (pleural)	Random phase 2/20.1	NIVO *vs*. NIVO + IPI	Pretreated	125	19 *vs*. 28	4.0 (2.8–5.7) *vs*. 5.6 (3.1–8.3)	11.9 (6.7–17.7) *vs*. 15.9 (10.7–nr)	Stomatitis, artritis, AST, ALT ↑
***Moderately*–*poorly responsive tumors (response rate <20%; median PFS <3*–*6 months; median OS <12*–*24 months)***									
***head and neck tumors***									
**Rodriguez/2020** (41)	Salivary gland	Phase 2/13.1	PEMBRO + vorinostat	Advanced	25	16	6.9 (4.1–nr)	14.0 (8.5–nr)	Creatinine ↑, fatigue
**Mahmood/2021** (64)	Salivary gland	Phase 2/19.8	PEMBRO ± RT	Advanced	20	0	4.5 (2.4–20.6) *vs*. 6.6 (2.4–13.1)	nr *vs*. 27.2 (22.9–nr)	NR
***Sarcomas***									
**Ben-Hami/2017** (7)	Sarcoma (uterine)	Phase 2/–	NIVO	Pretreated	12	0	1.8 (0.8–nr)	nr	Reported only rare SAEs
**D’Angelo/2018** (53)	STS	Random phase 2/13.6	NIVO *vs*. NIVO + IPI	Pretreated	85	5 *vs*. 16	1.7 (1.4–4.3) *vs*. 4.1 (2.6–4.7)	10.7 (5.5–15.4) *vs.* 14.3 (9.6–nr)	Anorexia, fatigue; dyspnoea
**Kelly/2020** (60)	STS	Phase 2/14	PEMBRO + T–VEC	Pretreated	20	30	4.1 (3.0–nr)	18.6 (12.2–nr)	NR
**Le Cesne/2019** (23)	Osteosarcoma	Phase 2/18.9	PEMBRO + mCTX	Pretreated	17	6.7	1.4 (1.0–1.4)	5.6 (2.1–12.1)	Nausea, anaemia, fatigue
**Maki/2013** (25)	Synovial sarcoma	Phase 2/–	IPI	2nd	6	0	1.8 (0.4–2.1) TTP	8.7 (0.7–19.7)	Alkaline phosphatase, bilirubin ↑
**Tamura/2019** (4)	STS	Phase 2/10.2	NIVO	Pretreated	21	0	1.4 (1.4–2.8)	ne (10.8–ne)	Pruritus, hypothyroidism, AST, ALT ↑
**Tawbi/2017** (44)	STS Bone sarcoma	Phase 2/17.8	PEMBRO	Pretreated	80	18 5	4.5 (2.0–5.2) 2.0 (1.7–2.2)	12.2 (8.5–18.2) 13.0 (10.0–18.0)	Only rare SAE reported
**Toulmonde/2018** (45)	STS (various)	Phase 2/6.8	PEMBRO + mCTX	Advanced	50	2	1.4 (1.2–1.4) *vs.* 1.4 (1.1–4.0) *vs.* 1.4 (0.9–4.0) *vs.* 1.4 (0.9–5.3)**	9.2 (2.4–15.9) *vs.* 5.6 (3.2–16.1) *vs.* 7.1 (2.0–16.3) *vs.* nr**	NR
**Wilky/2019** (47)	STS	Phase 2/14.7	PEMBRO + axitinib	Pretreated	33	25	4.7 (3.0–9.4)	18.7 (12.0–nr)	Fatigue, mucositis, thyroid disfunction
***Male urological cancers***									
**Adra/2018** (6)	Germ-cell	Phase 2/–	PEMBRO	Pretreated	12	0	–	–	Fatigue, nausea, vomiting
**Mego/2019** (27)	Germ-cell	Phase 2/2.6	AVE	Pretreated	8	0	0.9 (0.5–1.9)	2.7 (1.0–3.3)	Pain (G3)
**Necchi/2019** (31)	Germ-cell	Phase 2/7.5	DURVA *vs.* DURVA + TREME	Advanced	22	9.1	–	–	NR
***Endocrine and neuroendocrine tumors***									
**Capdevila/2020** (58)	Thyroid (anaplastic)	Phase 2/–	SPARTA	Advanced	42	19	1.7 (1.2–1.9)	5.9 (2.4–nr)	Diarrhoea, pruritus, fatigue
**Carneiro/2019** (55)	Adrenocortical carcinoma	Phase 2/–	NIVO	Pretreated	10	10	1.8 (0.1–4.3)	21.2 (0.1–>25.6)	Rash, fatigue
**Chintakuntlawar/2019** (48)	Thyroid (anaplastic)	Phase 2/–	CTRT + PEMBRO	1st	3	–	–	2.7 (–)	Pneumonitis
**Habra/2019** (13)	Adrenocortical carcinoma	Phase 2/–	PEMBRO	Pretreated	14	14	–	–	Fatigue, rash, hypothyroidism
**Klein/2020** (56)	NET	Phase 2/–	IPI + NIVO	Pretreated	29	24	4.8 (2.7–10.5)	14.8 (4.1–21.3)	NR
**LeTourneau/2018** (66)	Adrenocortical carcinoma	Phase 2/–	AVE	Pretreated	50	6	2.6 (1.4–4.0)	10.6 (7.4–15)	Nausea, fatigue, fever
**Mehnert/2019** (59)	Thyroid (papillary/follicular)	Phase 1b/31	PEMBRO	Pretreated	21	9	7.0 (2.0–14.0)	nr (22.0–nr)	Diarrhoea, fatigue, pruritus, rash
**Jimenez/2020** (14)	Adrenocortical carcinoma	Phase 2/–	PEMBRO	Pretreated	15	15	–	–	AST, ALT and alkaline phosphatase ↑
**Naing/2020** (5)	Pheochromocytomas/paragangliomas	Phase 2/–	PEMBRO	Pretreated	9	0	–	–	Fatigue, rash, hypothiroidism
**Patel/2020** (36)	Non-pancreatic NET	Phase 2/–	IPI + NIVO	Pretreated	32	25	4.0 (3.0–6.0)	11.0 (6.0–nr)	Fatigue, nausea, vomiting
**Rai/2019** (40)	Adrenocortical carcinoma	Phase 2/17.8	PEMBRO	Advanced	39	23	2.1 (2.0–10.7)	24.9 (4.2–nr)	AST, ALT ↑, fatigue
**Vijayvergia/2020** (46)	NET	Phase 2/–	PEMBRO	Pretreated	29	3.4	2.2 (1.5–2.3)	5.1 (3.2–ne)	AST, alkaline phosphatase ↑, fatigue

ORR, overall response rate; PFS, progression-free survival; TTP, time to progression; OS, overall survival; CI, confidence interval; SCC, cutaneous squamous cell carcinoma; HCC, hepatocellular carcinoma; NSCLC, non-small-cell lung cancer; STS, soft-tissue sarcoma; NET, neuroendocrine tumor; AVE, avelumab; NIVO, nivolumab; IPI, ipilimumab; PEMBRO, pembrolizumab; TREME, tremelimumab; CEMI, cemiplimab; ATEZO, atezolimumab; DURVA, durvalumab; SPARTA, spartalizumab; BEV, bevacizumab; CTRT, chemoradiotherapy; CDDP, cisplatin; GEM, gemcitabine; PEME, pemetrexed; mCTX, metronomic cyclophosphamide; T-VEC, talimogene laherparepvec; RT, radiotherapy; BSC, best supportive care; nr, not reached; ne, not estimable; –, not reported.

*Thymic carcinoma.

**Three different sarcoma subgroups

°By central review assessment.

^Noninfected patients. ↑increase the other: followed by.

### Skin Cancers

Most data on the use of ICIs in skin cancer were derived from studies on SCCs [n = 4 studies (5, 26, 29, 30)] and MCCs [n = 3 studies (19, 32, 53)]. The ORRs were >40% and 60%, respectively, when ICIs were used as first-line treatment in both cancers. Survival data were from the early stages, or median outcomes had not been reached. In these two settings, ICIs largely replaced the previous standard of care in locoregional or distant relapses (e.g., radiotherapy or cisplatin-based chemotherapy).

Immunotherapy was also explored in extracutaneous melanomas, such as mucosal or uveal melanomas (n = 7 studies). Data from a pooled analysis confirmed fair activity in mucosal melanoma of nivolumab alone or with ipilimumab (ORR of 23% and 37%, respectively). Conversely, ICIs demonstrated limited activity in uveal melanoma, with a median PFS of a few months (61).

### Hepatobiliary Cancers

Hepatocellular carcinoma (HCC) has been a popular target for ICI therapy investigations over the last years. At least four phase 2 studies explored pembrolizumab and nivolumab after first-line failure (sorafenib), with a mean ORR, median PFS, and OS of about 20% (range, 18–22%), 4.5 months (range, 3–6.9), and 12.2 months (range, 8.2–13.9), respectively (10, 39, 42, 49). A phase 3 study also established atezolizumab + bevacizumab, the new standard first-line therapy in advanced HCC (11). Biliary tract cancers (BTCs) were also included in phase 2 studies investigating ICIs; however, preliminary data were unsatisfactory, with few response rates and median PFS and OS not reported (9, 17, 21, 22).

### Thoracic Cancers

Eight phase 2 trials (n = 734 patients) evaluated ICIs in pleural mesothelioma (MPM) (8, 24, 28, 34, 35, 37, 38, 43). Only two studies included ICIs as a first-line strategy. Two appropriate therapeutic options have shown promising activity in MPM: the anti-programmed cell death protein 1 (PD-1) antibodies pembrolizumab and nivolumab as single agents, and nivolumab with ipilimumab. The Food and Drug Administration (FDA) has approved nivolumab + ipilimumab for unresectable mesothelioma; the ORR was 29% with nivolumab and ipilimumab. As a first-line approach, a combination of ICIs (anti-PD1 and anti-CTLA4) was associated with a median OS of 16.6 months. The combination of durvalumab plus cisplatin and pemetrexed demonstrated an ORR of 48% and an OS of 18.4 months. A confirmatory phase 3 study established the combination of nivolumab + ipilimumab as a potential new standard of care for previously untreated patients with MPM (63).

The role of ICIs was also explored in pretreated thymic epithelial tumours. Pembrolizumab showed fair activity, with an ORR of about 20% (12, 51), while no apparent activity was associated with nivolumab (18).

### Sarcomas

Nine phase 2 studies evaluated ICIs in 324 patients with advanced pretreated sarcomas (including uterine sarcomas and bone sarcomas) (4, 7, 23, 25, 44, 45, 47, 53, 60). ORRs were rare overall, and the median PFS was approximately 1–2 months; however, three trials reported an ORR >10% and a median PFS of more than 3 months. Among the latter, in a combination trial of pembrolizumab and axitinib (n = 33 patients), the ORR was 25% and median PFS and OS were 4.7 (range, 3.0–9.4) and 18.7 [range, 12.0 to not reached (NR)] months, respectively (47). In another trial of pembrolizumab and T-VEC (n = 20 patients), the ORR was 30%, and median PFS and OS were 4.1 (3.0 to NR) and 18.6 (12.2 to NR) months, respectively (60).

### Endocrine Cancers

Eleven trials evaluated ICIs in 215 patients with endocrine malignancies, including adrenocortical carcinomas (ACC, n = 5), neuroendocrine tumours (NETs, n = 3), thyroid carcinoma (n = 2), and pheochromocytomas/paragangliomas (PCPG, n = 1) (5, 36, 40, 46, 48, 55, 56, 58, 59).

In the ACC setting, three ICI agents (i.e., avelumab, nivolumab, and pembrolizumab) were evaluated in pretreated patients. ORR ranged from 6% to 23%. Median PFS was below 3 months, and OS ranged from 21 to 24 months. Nine patients with pheochromocytomas/paragangliomas were treated as a subcohort in one trial with pembrolizumab. No responses were observed, and the median PFS and OS were 5.7 and 19 months, respectively.

Three studies explored the role of ICIs in 90 patients with NETs. Anti-PD1 monotherapy with pembrolizumab was associated with lower ORRs than combo-immunotherapy with nivolumab-ipilimumab (3.4% versus 25%).

Only three patients with anaplastic carcinoma of the thyroid were included in a phase 2 trial of chemo-immunotherapy with pembrolizumab. No responses were observed, and the median OS was <3 months.

### Other Cancer Types

Salivary gland carcinomas are putative targets for ICIs (41). In a phase 2 trial, pembrolizumab + vorinostat was associated with a median OS of 14 months (ORR of 16%). No activity was reported with ICI in anaplastic or differentiated thyroid cancer or with pembrolizumab + radiotherapy in adenoid cystic carcinoma.

### Correlation of ORR With OS

**Figure 2:**
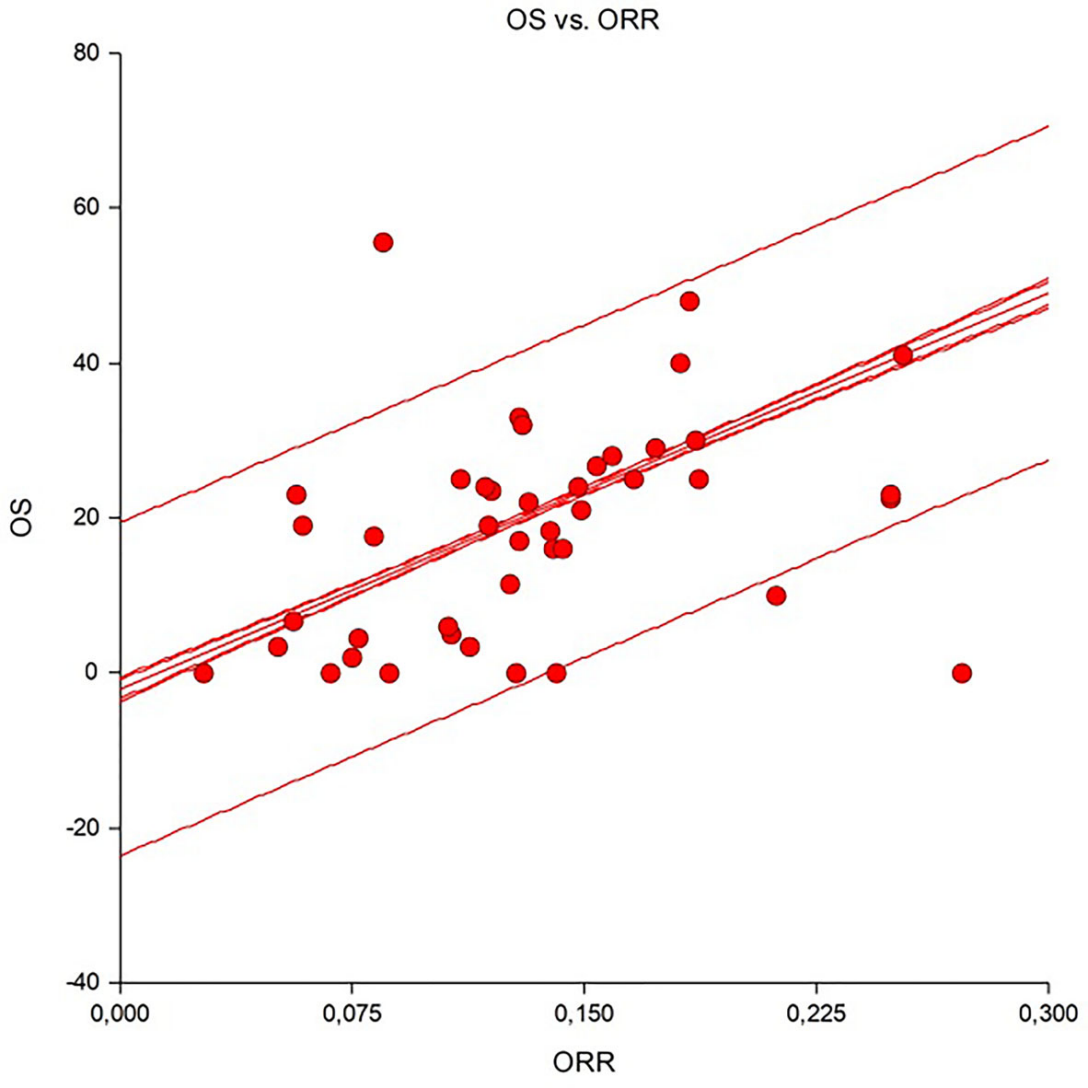
Linear correlation between overall response rate and overall survival in studies analysed.

A significant and good linear correlation was observed between the ORR and OS (**Figure 2**). The Spearman correlation coefficient was 0.69, indicating that about 70% of outcomes could be driven by tumour response. According to the calculations for various diseases, this correlation was very high for skin cancers (R^2^ = 0.98), mesotheliomas (R^2^ = 0.97), and sarcomas (R^2^ = 0.93), moderate for endocrine neoplasm (R^2^ = 0.65) and poor (R^2^ = −0.14) for hepatobiliary cancers. This correlation was similar both in highly responsive disease (e.g., skin cancers) and in hard-to-treat cancers (e.g., sarcomas, head and neck or endocrine neoplasm) where R^2^ were 0.7 and 0.66, respectively. This means that disease shrinkage may be useful when drug are screened for rare tumours clinical trials.

## Discussion

In this study, we conducted a systematic review of published studies with full-text outcome data that explored the efficacy of ICIs in rare solid tumours.

More than 60 trials with different ICIs in rare cancers were conducted over 7 years between 2013 and 2021, with most trials published in the last 3 years (2018–2021). Our search identified 17 groups of rare neoplasms treated in most cases in patients with advanced and pretreated diseases. Our principal objective was to determine the objective response rate in this setting rather than the survival endpoints that are influenced by several variables for each neoplasm. It is worth noting that most of the studies included here were small phase 2 trials in which OS was not reached or the primary endpoint.

A first observation from our analysis is that the ORR varied widely, from 0% to 60%, with a slightly lower median rate of 20% among all groups of rare cancers. This indicates that the ORR obtained with ICIs in rare cancers does not significantly differ from that of more common neoplasms. Among the rare cancer types that displayed response rates higher than 25% were MCC, SCC of the skin, mesothelioma, BTCs, and some NETs.

The analysis of survival endpoints was not as informative as that of ORR. This was partly because these data were not based on the primary endpoints of the corresponding trials or were not reported. In general, PFS did not significantly exceed 6 months in all trials and all groups (except two trials in MCC), with the poorest PFS rates observed in patients with sarcomas, germ cell tumours, and adrenal neoplasms, in which median PFS was below 3 months. Rates of PFS and OS were found to be aligned with those of each neoplasm in the corresponding setting and, therefore, reflect/represent more the intrinsic clinical course of each disease than definitive immune resistance. Regression of the ORR with OS showed a modest correlation, demonstrating that cancer shrinkage is not a prerequisite for longer survival.

Since the early observation that approximately 20% of patients with cancer respond to immunotherapy with ICIs (67), the prediction of each neoplasm’s responsiveness and each individual patients’ physiology have become central issues in immune oncology. Research initially focused on investigating single biomarkers or pathways potentially involved in response to ICIs, particularly the PD-1/programmed death-ligand 1 (PD-L1) axis, and the extent to which tumour-infiltrating lymphocytes (TILs) are present within cancer tissue (68–70). In the case of PD-L1, such an approach led regulatory authorities to approve the clinical use of ICIs in selected cancers (e.g., non-small cell lung cancer) under conditional expression of PD-L1 at different cutoff levels. By contrast, in other neoplasms (e.g., head and neck squamous cell carcinomas), approval was granted regardless of PD-L1 expression, indicating that clinical benefit could be obtained independently of PD-L1 expression or TIL infiltration grade (71).

Second-generation studies focused on tumour mutation burden (TMB) as a measure of the neoantigen load and, therefore, tumour foreignness within the immune system (72). Reclassification of tumours from the The Cancer Genome Atlas (TCGA) datasets did prove a clear relationship between TMB levels and the ORR obtained using ICIs (73). On this basis, new ICIs received approval in an agnostic way for TMB-high tumours (e.g., small cell lung cancer). However, very recent evidence calls for more caution in using high TMB as a universal predictive biomarker for all ICIs and in all cancers (74). In 2016, Blank et al. proposed an “immunogram” to create a framework of the multifaceted, dynamic interactions between cancers and the immune system (75). In line with this view, more recent studies have provided better predictive stratification by applying a three-key-variables analysis, which includes CD8+ T-cell abundance and TMB and PD-1 expression levels (76). Despite this strategy being superior to the single-marker strategy, it is still limited in its prediction capacity, indicating a higher complexity of the cancer-immunity cycle. Recently, a work by Wang et al. focused on immunotherapy response as a function of tumour immunogenicity (TIG), which is the result of tumour antigenicity (e.g., neoantigen load) and antigen presentation capacity. In this analysis, neoplasms with low TIG scores had low response rates to immunotherapy despite very significant antigenicity (77).

The above-cited studies offer a framework for analysing the results of the ICI immunotherapy trials in rare neoplasms cited in this systematic review. With the highest multiparameter TIG scores are cancers such as MCC and cutaneous SCC—all neoplasms with response rates higher than 30% and up to 62%. At the opposite end of the scale, with the lowest TIG scores, are some rare neoplasms, including ACC, PCPG, soft tissue sarcomas, uveal melanoma, germ cell tumours, and low-grade gliomas. These tumours come from anatomical sites that are considered “immune-privileged” and were all characterised by response rates well below 30% (and down to 0% in many cases) in several different trials. More common cancers, such as prostate and breast carcinomas, also segregate into this subgroup. The intermediate TIG score group includes rare neoplasms, such as BTCs, HCC, mesothelioma, and thymic carcinoma, and other more common neoplasms (e.g., lung, head and neck and renal cell cancers). In our analysis, the ORR of these neoplasms was in the range of 20–45%. This portrait is confirmed by our attempt to link OS with ORR, attaining a strong correlation in skin cancers, mesothelioma, and sarcomas. In these cancers, despite data about OS and ORR being available in only 43 studies, more than 90% of observed outcomes may be explained by a durable tumour response as observed in non-melanoma skin cancers and mesothelioma.

There are several limitations associated with our systematic review. First, only a few randomised studies that compared ICIs with standard treatments are available, and a direct comparison was not possible. Second, many confirmatory phase 3 studies are ongoing and were not included in the present review. Third, there was a lack of information regarding predictive biomarkers in many trials, so further knowledge is awaited from ongoing correlative studies.

Based on the present systematic review results and in the absence of final approval for most of these indications, the present data suggest that for some conditions (e.g., HCC, non-melanoma skin cancers and MCC), treatment with ICIs offers significant clinical benefit. In particular, for non-melanoma skin cancers, the activity of ICIs is outstanding, and cemiplimab was recently approved for advanced squamous cell histology in both the US and European countries. In other rare tumours, the efficacy of ICI therapy cannot be fully ascertained because of the small sample sizes and non-randomised design of clinical trials (78, 79).

In conclusion, these considerations raise some final questions: Is the prediction of response to ICIs in rare cancers different from that of common cancers? What are the minimum immunological predictive factors for selecting patients with different responses to ICI therapy in rare cancers? What kind of data should be collected from future trials of immunotherapy in rare solid cancers? At present, we can only attempt to address a limited number of points. First, mechanisms that regulate immune responses are universal and do not differ among rare and common cancers. However, the immunological environment in which specific cancers develop can be specific and diverse, regardless of their rarity (80, 81). For example, in ACC, several drivers of intrinsic immunoresistance have been identified, including alteration of the WNT/beta-catenin pathway, TP53 mutations, cortisol hypersecretion, and PD-L1 downregulation (82, 83). These elements derive from the specific genomic landscape of ACC and cannot be generalised to other cancers. Second, beyond clinical trials, the on-label prescription of immunotherapy is currently granted for a few rare cancers. In these limited cases, clinicians are asked to provide minimum predictive factors of immunoresponse, such as PD-L1 expression, TMB, or MSI status. New immunological markers require more validation and are not yet ready for routine implementation. Multiparameter analysis of biomarkers that are predictive of the potential activity of ICIs in rare cancers and in common cancers will open new avenues for better selection of patients who may benefit from immunotherapy.

## Data Availability Statement

The original contributions presented in the study are included in the article. Further inquiries can be directed to the corresponding author.

## Author Contributions

All authors listed have made a substantial, direct, and intellectual contribution to the work and approved it for publication.

## Conflict of Interest

The authors declare that the research was conducted in the absence of any commercial or financial relationships that could be construed as a potential conflict of interest.

## Publisher’s Note

All claims expressed in this article are solely those of the authors and do not necessarily represent those of their affiliated organizations, or those of the publisher, the editors and the reviewers. Any product that may be evaluated in this article, or claim that may be made by its manufacturer, is not guaranteed or endorsed by the publisher.

## References

[1] Finn RS Ryoo BY Merle P Kudo M Bouattour M Lim HY. Pembrolizumab As Second-Line Therapy in Patients With Advanced Hepatocellular Carcinoma in KEYNOTE-240: A Randomized, Double-Blind, Phase III Trial. J Clin Oncol (2020) 38(3):193–202. doi: 10.1200/JCO.19.01307 31790344

[2] Gatta G van der Zwan JM Casali PG Siesling S Dei Tos AP Kunkler I. Rare Cancers Are Not So Rare: The Rare Cancer Burden in Europe. Eur J Cancer (2011) 47(17):2493–511. doi: 10.1016/j.ejca.2011.08.008 22033323

[3] Komatsubara KM Carvajal RD. The Promise and Challenges of Rare Cancer Research. Lancet Oncol (2016) 17(2):136–8. doi: 10.1016/S1470-2045(15)00485-4 26868336

[4] Tamura K Hasegawa K Katsumata N Matsumoto K Mukai H Takahashi S. Efficacy and Safety of Nivolumab in Japanese Patients With Uterine Cervical Cancer, Uterine Corpus Cancer, or Soft Tissue Sarcoma: Multicenter, Open-Label Phase 2 Trial. Cancer Sci (2019) 110(9):2894–904. doi: 10.1111/cas.14148 PMC6726684 31348579

[5] Naing A Meric-Bernstam F Stephen B Karp DD Hajjar J Rodon Ahnert J. Phase 2 Study of Pembrolizumab in Patients With Advanced Rare Cancers. J Immunother Cancer (2020) 8(1):e000347. doi: 10.1136/jitc-2019-000347 32188704 PMC7078933

[6] Adra N Einhorn LH Althouse SK Ammakkanavar NR Musapatika D Albany C. Phase II Trial of Pembrolizumab in Patients With Platinum Refractory Germ-Cell Tumors: A Hoosier Cancer Research Network Study GU14-206. Ann Oncol (2018) 29(1):209–14. doi: 10.1093/annonc/mdx680 29045540

[7] Ben-Ami E Barysauskas CM Solomon S Tahlil K Malley R Hohos M. Immunotherapy With Single Agent Nivolumab for Advanced Leiomyosarcoma of the Uterus: Results of a Phase 2 Study. Cancer (2017) 123(17):3285–90. doi: 10.1002/cncr.30738 PMC5762200 28440953

[8] Disselhorst MJ Quispel-Janssen J Lalezari F Monkhorst K de Vries JF van der Noort V. Ipilimumab and Nivolumab in the Treatment of Recurrent Malignant Pleural Mesothelioma (INITIATE): Results of a Prospective, Single-Arm, Phase 2 Trial. Lancet Respir Med (2019) 7(3):260–70. doi: 10.1016/S2213-2600(18)30420-X 30660511

[9] Feng K Liu Y Zhao Y Yang Q Dong L Liu J. Efficacy and Biomarker Analysis of Nivolumab Plus Gemcitabine and Cisplatin in Patients With Unresectable or Metastatic Biliary Tract Cancers: Results From a Phase II Study. J Immunother Cancer (2020) 8(1):e000367. doi: 10.1136/jitc-2019-000367 32487569 PMC7269541

[10] Feun LG Li Y Wu C Wangpaichitr M Jones PD Richman SP. Phase 2 Study of Pembrolizumab and Circulating Biomarkers to Predict Anticancer Response in Advanced, Unresectable Hepatocellular Carcinoma. Cancer (2019) 125(20):3603–14. doi: 10.1002/cncr.32339.Phase PMC7592647 31251403

[11] Finn RS Qin S Ikeda M Galle PR Ducreux M Kim TY. Atezolizumab Plus Bevacizumab in Unresectable Hepatocellular Carcinoma. N Engl J Med (2020) 382(20):1894–905. doi: 10.1056/nejmoa1915745 32402160

[12] Giaccone G Kim C Thompson J McGuire C Kallakury B Chahine JJ. Pembrolizumab in Patients With Thymic Carcinoma: A Single-Arm, Single-Centre, Phase 2 Study. Lancet Oncol (2018) 19(3):347–55. doi: 10.1016/S1470-2045(18)30062-7 PMC10683856 29395863

[13] Habra MA Stephen B Campbell M Hess K Tapia C Xu M. Phase II Clinical Trial of Pembrolizumab Efficacy and Safety in Advanced Adrenocortical Carcinoma. J Immunother Cancer (2019) 7(1):1–9. doi: 10.1186/s40425-019-0722-x 31533818 PMC6751592

[14] Jimenez C Subbiah V Stephen B Ma J Milton D Xu M. Phase II Clinical Trial of Pembrolizumab in Patients With Progressive Metastatic Pheochromocytomas and Paragangliomas. Cancers (Basel) (2020) 12(8):1–15. doi: 10.3390/cancers12082307 PMC7465458 32824391

[15] Johnson DB Bao R Ancell KK Daniels AB Wallace D Sosman JA. Response to Anti–PD-1 in Uveal Melanoma Without High-Volume Liver Metastasis. JNCCN J Natl Compr Cancer Netw (2019) 17(2):114–7. doi: 10.6004/jnccn.2018.7070 PMC8063157 30787124

[16] Joshua AM Monzon JG Mihalcioiu C Hogg D Smylie M Cheng T. A Phase 2 Study of Tremelimumab in Patients With Advanced Uveal Melanoma. Melanoma Res (2015) 25(4):342–7. doi: 10.1097/CMR.0000000000000175 26050146

[17] Boilève A Hilmi M Gougis P Cohen R Rousseau B Blanc JF. Triplet Combination of Durvalumab, Tremelimumab, and Paclitaxel in Biliary Tract Carcinomas: Safety Run-in Results of the Randomized IMMUNOBIL PRODIGE 57 Phase II Trial. Eur J Cancer (2021) 143:55–63. doi: 10.1016/j.ejca.2020.10.027 33279854

[18] Katsuya Y Horinouchi H Seto T Umemura S Hosomi Y Satouchi M. Single-Arm, Multicentre, Phase II Trial of Nivolumab for Unresectable or Recurrent Thymic Carcinoma: PRIMER Study. Eur J Cancer (2019) 113:78–86. doi: 10.1016/j.ejca.2019.03.012 30991261

[19] Kaufman HL Russell JS Hamid O Bhatia S Terheyden P D'Angelo SP. Updated Efficacy of Avelumab in Patients With Previously Treated Metastatic Merkel Cell Carcinoma After ≥1 Year of Follow-Up: JAVELIN Merkel 200, a Phase 2 Clinical Trial. J Immunother Cancer (2018) 6(1):4–10. doi: 10.1186/s40425-017-0310-x 29347993 PMC5774167

[20] Kim M Keam B Ock CY Kim SH Kim YJ Lim SM. Phase II Study of Durvalumab and Tremelimumab in Pulmonary Sarcomatoid Carcinoma: KCSG-LU16-07. Thorac Cancer (2020) 11(12):3482–9. doi: 10.1111/1759-7714.13684 PMC7705626 33026712

[21] Kim RD Chung V Alese OB El-Rayes BF Li D Al-Toubah TE. A Phase 2 Multi-Institutional Study of Nivolumab for Patients With Advanced Refractory Biliary Tract Cancer. JAMA Oncol (2020) 6(6):888–94. doi: 10.1001/jamaoncol.2020.0930 PMC7193528 32352498

[22] Klein O Kee D Nagrial A Markman B Underhill C Michael M. Evaluation of Combination Nivolumab and Ipilimumab Immunotherapy in Patients With Advanced Biliary Tract Cancers: Subgroup Analysis of a Phase 2 Nonrandomized Clinical Trial. JAMA Oncol (2020) 6(9):1405–9. doi: 10.1001/jamaoncol.2020.2814 PMC7393585 32729929

[23] Le Cesne A Marec-Berard P Blay JY Gaspar N Bertucci F Penel N. Programmed Cell Death 1 (PD-1) Targeting in Patients With Advanced Osteosarcomas: Results From the PEMBROSARC Study. Eur J Cancer (2019) 119(August):151–7. doi: 10.1016/j.ejca.2019.07.018 31442817

[24] Maio M Scherpereel A Calabrò L Aerts J Perez SC Bearz A. Tremelimumab as Second-Line or Third-Line Treatment in Relapsed Malignant Mesothelioma (DETERMINE): A Multicentre, International, Randomised, Double-Blind, Placebo-Controlled Phase 2b Trial. Lancet Oncol (2017) 18(9):1261–73. doi: 10.1016/S1470-2045(17)30446-1 28729154

[25] Maki RG Jungbluth AA Gnjatic S Schwartz GK D'Adamo DR Keohan ML. A Pilot Study of Anti-CTLA4 Antibody Ipilimumab in Patients With Synovial Sarcoma. Sarcoma (2013) 2013:168145. doi: 10.1155/2013/168145 23554566 PMC3608267

[26] Maubec E Boubaya M Petrow P Beylot-Barry M Basset-Seguin N Deschamps L. Phase II Study of Pembrolizumab as First-Line, Single-Drug Therapy for Patients With Unresectable Cutaneous Squamous Cell Carcinomas. J Clin Oncol (2020) 38(26):3051–61. doi: 10.1200/JCO.19.03357 32730186

[27] Mego M Svetlovska D Chovanec M Rečkova M Rejlekova K Obertova J. Phase II Study of Avelumab in Multiple Relapsed/Refractory Germ Cell Cancer. Invest New Drugs (2019) 37(4):748–54. doi: 10.1007/s10637-019-00805-4 31152292

[28] Calabrò L Morra A Fonsatti E Cutaia O Fazio C Annesi D. Efficacy and Safety of an Intensified Schedule of Tremelimumab for Chemotherapy-Resistant Malignant Mesothelioma: An Open-Label, Single-Arm, Phase 2 Study. Lancet Respir Med (2015) 3(4):301–9. doi: 10.1016/S2213-2600(15)00092-2 25819643

[29] Migden MR Khushalani NI Chang ALS Lewis KD Schmults CD Hernandez-Aya L. Cemiplimab in Locally Advanced Cutaneous Squamous Cell Carcinoma: Results From an Open-Label, Phase 2, Single-Arm Trial. Lancet Oncol (2020) 21(2):294–305. doi: 10.1016/S1470-2045(19)30728-4 31952975 PMC7771329

[30] Migden MR Rischin D Schmults CD Guminski A Hauschild A Lewis KD. PD-1 Blockade With Cemiplimab in Advanced Cutaneous Squamous-Cell Carcinoma. N Engl J Med (2018) 379(4):341–51. doi: 10.1056/nejmoa1805131 29863979

[31] Necchi A Giannatempo P Raggi D Mariani L Colecchia M Farè E. An Open-Label Randomized Phase 2 Study of Durvalumab Alone or in Combination With Tremelimumab in Patients With Advanced Germ Cell Tumors (APACHE): Results From the First Planned Interim Analysis. Eur Urol (2019) 75(1):201–3. doi: 10.1016/j.eururo.2018.09.010 30243800

[32] Nghiem P Bhatia S Lipson EJ Sharfman WH Kudchadkar RR Brohl AS. Durable Tumor Regression and Overall Survival in Patients With Advanced Merkel Cell Carcinoma Receiving Pembrolizumab as First-Line Therapy. J Clin Oncol (2019) 37(9):693–702. doi: 10.1200/JCO.18.01896 30726175 PMC6424137

[33] Nomura M Oze I Masuishi T Yokota T Satake H Iwasawa S. Multicenter Prospective Phase II Trial of Nivolumab in Patients With Unresectable or Metastatic Mucosal Melanoma. Int J Clin Oncol (2020) 25(5):972–7. doi: 10.1007/s10147-020-01618-9 31938955

[34] Nowak AK Lesterhuis WJ Kok PS Brown C Hughes BG Karikios DJ. Durvalumab With First-Line Chemotherapy in Previously Untreated Malignant Pleural Mesothelioma (DREAM): A Multicentre, Single-Arm, Phase 2 Trial With a Safety Run-in. Lancet Oncol (2020) 21(9):1213–23. doi: 10.1016/S1470-2045(20)30462-9 32888453

[35] Okada M Kijima T Aoe K Kato T Fujimoto N Nakagawa K. Clinical Efficacy and Safety of Nivolumab: Results of a Multicenter, Open-Label, Single-Arm, Japanese Phase II Study in Malignant Pleural Mesothelioma (MERIT). Clin Cancer Res (2019) 25(18):5485–92. doi: 10.1158/1078-0432.CCR-19-0103 31164373

[36] Patel SP Othus M Chae YK Giles FJ Hansel DE Singh PP. A Phase II Basket Trial of Dual Anti–CTLA-4 and Anti–PD-1 Blockade in Rare Tumors (DART SWOG 1609) in Patients With Nonpancreatic Neuroendocrine Tumors. Clin Cancer Res (2020) 26(10):2290–6. doi: 10.1158/1078-0432.CCR-19-3356 PMC7231627 31969335

[37] Quispel-Janssen J van der Noort V de Vries JF Zimmerman M Lalezari F Thunnissen E. Programmed Death 1 Blockade With Nivolumab in Patients With Recurrent Malignant Pleural Mesothelioma. J Thorac Oncol (2018) 13(10):1569–76. doi: 10.1016/j.jtho.2018.05.038 29908324

[38] Calabrò L Morra A Giannarelli D Amato G D'Incecco A Covre A. Tremelimumab Combined With Durvalumab in Patients With Mesothelioma (NIBIT-MESO-1): An Open-Label, Non-Randomised, Phase 2 Study. Lancet Respir Med (2018) 6(6):451–60. doi: 10.1016/S2213-2600(18)30151-6 29773326

[39] Quispe-Tintaya W. 乳鼠心肌提取 HHS Public Access. Physiol Behav (2017) 176(3):139–48. doi: 10.1016/S0140-6736(17)31046-2.Nivolumab

[40] Raj N Zheng Y Kelly V Katz SS Chou J Do RKG. PD-1 Blockade in Advanced Adrenocortical Carcinoma. J Clin Oncol (2020) 38(1):71–80. doi: 10.1200/JCO.19.01586 31644329 PMC7351334

[41] Rodriguez CP Wu Q Voutsinas J Fromm JR Jiang X Pillarisetty VG. A Phase II Trial of Pembrolizumab and Vorinostat in Recurrent Metastatic Head and Neck Squamous Cell Carcinomas and Salivary Gland Cancer. Clin Cancer Res (2020) 26(4):837–45. doi: 10.1158/1078-0432.CCR-19-2214 31796519

[42] Sangro B Gomez-Martin C de la Mata M Iñarrairaegui M Garralda E Barrera P. A Clinical Trial of CTLA-4 Blockade With Tremelimumab in Patients With Hepatocellular Carcinoma and Chronic Hepatitis C. J Hepatol (2013) 59(1):81–8. doi: 10.1016/j.jhep.2013.02.022 23466307

[43] Scherpereel A Mazieres J Greillier L Lantuejoul S Dô P Bylicki O. Nivolumab or Nivolumab Plus Ipilimumab in Patients With Relapsed Malignant Pleural Mesothelioma (IFCT-1501 MAPS2): A Multicentre, Open-Label, Randomised, Non-Comparative, Phase 2 Trial. Lancet Oncol (2019) 20(2):239–53. doi: 10.1016/S1470-2045(18)30765-4 30660609

[44] Tawbi HA Burgess M Bolejack V Van Tine BA Schuetze SM Hu J. Pembrolizumab in Advanced Soft-Tissue Sarcoma and Bone Sarcoma (SARC028): A Multicentre, Two-Cohort, Single-Arm, Open-Label, Phase 2 Trial. Lancet Oncol (2017) 18(11):1493–501. doi: 10.1016/S1470-2045(17)30624-1 PMC7939029 28988646

[45] Toulmonde M Penel N Adam J Chevreau C Blay JY Le Cesne A. Use of PD-1 Targeting, Macrophage Infiltration, and IDO Pathway Activation in Sarcomas a Phase 2 Clinical Trial. JAMA Oncol (2018) 4(1):93–7. doi: 10.1001/jamaoncol.2017.1617 PMC5833654 28662235

[46] Vijayvergia N Dasari A Deng M Litwin S Al-Toubah T Alpaugh RK. Pembrolizumab Monotherapy in Patients With Previously Treated Metastatic High-Grade Neuroendocrine Neoplasms: Joint Analysis of Two Prospective, non-Randomised Trials. Br J Cancer (2020) 122(9):1309–14. doi: 10.1038/s41416-020-0775-0 PMC7188798 32152503

[47] Wilky BA Trucco MM Subhawong TK Florou V Park W Kwon D. Axitinib Plus Pembrolizumab in Patients With Advanced Sarcomas Including Alveolar Soft-Part Sarcoma: A Single-Centre, Single-Arm, Phase 2 Trial. Lancet Oncol (2019) 20(6):837–48. doi: 10.1016/S1470-2045(19)30153-6 31078463

[48] Chintakuntlawar AV Yin J Foote RL Kasperbauer JL Rivera M Asmus E. A Phase 2 Study of Pembrolizumab Combined With Chemoradiotherapy as Initial Treatment for Anaplastic Thyroid Cancer. Thyroid (2019) 29(11):1615–22. doi: 10.1089/thy.2019.0086 31595822

[49] Zhu AX Finn RS Edeline J Cattan S Ogasawara S Palmer D. Pembrolizumab in Patients With Advanced Hepatocellular Carcinoma Previously Treated With Sorafenib (KEYNOTE-224): A Non-Randomised, Open-Label Phase 2 Trial. Lancet Oncol (2018) 19(7):940–52. doi: 10.1016/S1470-2045(18)30351-6 29875066

[50] Zimmer L Vaubel J Mohr P Hauschild A Utikal J Simon J. Phase II DeCOG-Study of Ipilimumab in Pretreated and Treatment-Naïve Patients With Metastatic Uveal Melanoma. PloS One (2015) 10(3):1–13. doi: 10.1371/journal.pone.0118564 PMC4356548 25761109

[51] Cho J Kim HS Ku BM Choi YL Cristescu R Han J. Pembrolizumab for Patients With Refractory or Relapsed Thymic Epithelial Tumor: An Open-Label Phase II Trial. J Clin Oncol (2019) 37(24):2162–70. doi: 10.1200/JCO.2017.77.3184 29906252

[52] D’Angelo SP Larkin J Sosman JA Lebbé C Brady B Neyns B. Efficacy and Safety of Nivolumab Alone or in Combination With Ipilimumab in Patients With Mucosal Melanoma: A Pooled Analysis. J Clin Oncol (2017) 35(2):226–35. doi: 10.1200/JCO.2016.67.9258 PMC5559888 28056206

[53] D’Angelo SP Mahoney MR Van Tine BA Atkins J Milhem MM Jahagirdar BN. Nivolumab With or Without Ipilimumab Treatment for Metastatic Sarcoma (Alliance A091401): Two Open-Label, Non-Comparative, Randomised, Phase 2 Trials. Lancet Oncol (2018) 19(3):416–26. doi: 10.1016/S1470-2045(18)30006-8 PMC6126546 29370992

[54] D’Angelo SP Russell J Lebbé C Chmielowski B Gambichler T Grob JJ. Efficacy and Safety of First-Line Avelumab Treatment in Patients With Stage IV Metastatic Merkel Cell Carcinoma a Preplanned Interim Analysis of a Clinical Trial. JAMA Oncol (2018) 4(9):1–5. doi: 10.1001/jamaoncol.2018.0077 29566106 PMC5885245

[55] Carneiro BA Konda B Costa RB Costa RLB Sagar B Gursel DB. Nivolumab in Metastatic Adrenocortical Carcinoma: Results of a Phase 2 Trial. J Clin Endocrinol Metab (2019) 104(12):6193–200. doi: 10.1210/jc.2019-00600 31276163

[56] Klein O Kee D Markman B Michael M Underhill C Carlino MS. Immunotherapy of Ipilimumab and Nivolumab in Patients With Advanced Neuroendocrine Tumors: A Subgroup Analysis of the CA209-538 Clinical Trial for Rare Cancers. Clin Cancer Res (2020) 26(17):4454–9. doi: 10.1158/1078-0432.CCR-20-0621 32532787

[57] Keilholz U Mehnert JM Bauer S Bourgeois H Patel MR Gravenor D. Avelumab in Patients With Previously Treated Metastatic Melanoma: Phase 1b Results From the JAVELIN Solid Tumor Trial. J Immunother Cancer (2019) 7(1):1–9. doi: 10.1186/s40425-018-0459-y 30651126 PMC6335739

[58] Capdevila J Wirth LJ Ernst T Ponce Aix S Lin CC Ramlau R. PD-1 Blockade in Anaplastic Thyroid Carcinoma. J Clin Oncol (2020) 38(23):2620–7. doi: 10.1200/JCO.19.02727 PMC7476256 32364844

[59] Mehnert JM Varga A Brose MS Aggarwal RR Lin CC Prawira A. Safety and Antitumor Activity of the Anti-PD-1 Antibody Pembrolizumab in Patients With Advanced, PD-L1-Positive Papillary or Follicular Thyroid Cancer. BMC Cancer (2019) 19(1):1–9. doi: 10.1186/s12885-019-5380-3 30832606 PMC6399859

[60] Kelly CM Antonescu CR Bowler T Munhoz R Chi P Dickson MA. Objective Response Rate Among Patients With Locally Advanced or Metastatic Sarcoma Treated With Talimogene Laherparepvec in Combination With Pembrolizumab: A Phase 2 Clinical Trial. JAMA Oncol (2020) 6(3):402–8. doi: 10.1001/jamaoncol.2019.6152 PMC6990941 31971541

[61] Schadendorf D Ascierto PA Haanen J Espinosa E Demidov L Garbe C. Safety and Efficacy of Nivolumab in Challenging Subgroups With Advanced Melanoma Who Progressed on or After Ipilimumab Treatment: A Single-Arm, Open-Label, Phase II Study (CheckMate 172). Eur J Cancer (2019) 121:144–53. doi: 10.1016/j.ejca.2019.08.014 31581055

[62] Nathan P Ascierto PA Haanen J Espinosa E Demidov L Garbe C. Safety and Efficacy of Nivolumab in Patients With Rare Melanoma Subtypes Who Progressed on or After Ipilimumab Treatment: A Single-Arm, Open-Label, Phase II Study (CheckMate 172). Eur J Cancer (2019) 119:168–78. doi: 10.1016/j.ejca.2019.07.010 31445199

[63] Baas P Scherpereel A Nowak AK Fujimoto N Peters S Tsao AS. First-Line Nivolumab Plus Ipilimumab in Unresectable Malignant Pleural Mesothelioma (CheckMate 743): A Multicentre, Randomised, Open-Label, Phase 3 Trial. Lancet (2021) 397(10272):375–86. doi: 10.1016/S0140-6736(20)32714-8 33485464

[64] Mahmood U Bang A Chen YH Mak RH Lorch JH Hanna GJ. A Randomized Phase 2 Study of Pembrolizumab With or Without Radiation in Patients With Recurrent or Metastatic Adenoid Cystic Carcinoma. Int J Radiat Oncol Biol Phys (2021) 109(1):134–44. doi: 10.1016/j.ijrobp.2020.08.018 PMC9361179 32781104

[65] El-Khoueiry AB Sangro B Yau T Crocenzi TS Kudo M Hsu C. Nivolumab in Patients With Advanced Hepatocellular Carcinoma (CheckMate 040): An Open-Label, Non-Comparative, Phase 1/2 Dose Escalation and Expansion Trial. Lancet (2017) 389(10088):2492–502. 10.1016/S0140-6736(17)31046-2 PMC7539326 28434648

[66] Le Tourneau C Hoimes C Zarwan C Wong DJ Bauer S Claus R. Avelumab in Patients With Previously Treated Metastatic Adrenocortical Carcinoma: Phase 1b Results From the JAVELIN Solid Tumor Trial. J Immunother Cancer (2018) 6(1):111. 30348224 10.1186/s40425-018-0424-9 PMC6198369

[67] Brahmer JR Drake CG Wollner I Powderly JD Picus J Sharfman WH. Phase I Study of Single-Agent Anti-Programmed Death-1 (MDX-1106) in Refractory Solid Tumors: Safety, Clinical Activity, Pharmacodynamics, and Immunologic Correlates. J Clin Oncol (2010) 28(19):3167–75. doi: 10.1200/JCO.2009.26.7609 PMC4834717 20516446

[68] Patel SP Kurzrock R. PD-L1 Expression as a Predictive Biomarker in Cancer Immunotherapy. Mol Cancer Ther (2015) 14(4):847–56. doi: 10.1158/1535-7163.MCT-14-0983 25695955

[69] Barnes TA Amir E. HYPE or HOPE: The Prognostic Value of Infiltrating Immune Cells in Cancer. Br J Cancer (2017) 118(2):e5. doi: 10.1038/bjc.2017.220 PMC5785752 29315291

[70] Roufas C Chasiotis D Makris A Efstathiades C Dimopoulos C Zaravinos A. The Expression and Prognostic Impact of Immune Cytolytic Activity-Related Markers in Human Malignancies: A Comprehensive Meta-Analysis. Front Oncol (2018) 8:27. doi: 10.3389/fonc.2018.00027 29515971 PMC5826382

[71] Davis AA Patel VG. The Role of PD-L1 Expression as a Predictive Biomarker: An Analysis of All US Food and Drug Administration (FDA) Approvals of Immune Checkpoint Inhibitors. J Immunother Cancer (2019) 7(1):278. doi: 10.1186/s40425-019-0768-9 31655605 PMC6815032

[72] Chabanon RM Pedrero M Lefebvre C Marabelle A Soria JC Postel-Vinay S. Mutational Landscape and Sensitivity to Immune Checkpoint Blockers. Clin Cancer Res (2016) 22(17):4309–21. doi: 10.1158/1078-0432.CCR-16-0903 27390348

[73] Yarchoan M Hopkins A Jaffee EM. Tumor Mutational Burden and Response Rate to PD-1 Inhibition. N Engl J Med (2017) 377(25):2500–501. doi: 10.1056/nejmc1713444 PMC6549688 29262275

[74] McGrail DJ Pilié PG Rashid NU Voorwerk L Slagter M Kok M. High Tumor Mutation Burden Fails to Predict Immune Checkpoint Blockade Response Across All Cancer Types. Ann Oncol (2021) 32(5):661–72. doi: 10.1016/j.annonc.2021.02.006 PMC8053682 33736924

[75] Blank CU Haanen JB Ribas A Schumacher TN. The “Cancer Immunogram.” Sci (80- ) (2016) 352(6286):658–60. doi: 10.1126/science.aaf2834 27151852

[76] Lee JS Ruppin E. Multiomics Prediction of Response Rates to Therapies to Inhibit Programmed Cell Death 1 and Programmed Cell Death 1 Ligand 1. JAMA Oncol (2019) 5(11):1614–8. doi: 10.1001/jamaoncol.2019.2311 PMC6707018 31436822

[77] Wang S He Z Wang X Li H Liu XS. Antigen Presentation and Tumor Immunogenicity in Cancer Immunotherapy Response Prediction. Elife (2019) 8:e49020. doi: 10.7554/eLife.49020 31767055 PMC6879305

[78] Vokinger KN Kesselheim AS. Application of Orphan Drug Designation to Cancer Treatments (2008-2017): A Comprehensive and Comparative Analysis of the USA and EU. BMJ Open (2019) 9(10):e028634. doi: 10.1136/bmjopen-2018-028634 PMC6797305 31601584

[79] Franco P. Orphan Drugs: The Regulatory Environment. Drug Discov Today (2013) 18(3–4):163–72. doi: 10.1016/j.drudis.2012.08.009 22981668

[80] Gatta G Capocaccia R Botta L Mallone S De Angelis R Ardanaz E. Burden and Centralised Treatment in Europe of Rare Tumours: Results of RARECAREnet—A Population-Based Study. Lancet Oncol (2017) 18(8):1022–39. doi: 10.1016/S1470-2045(17)30445-X 28687376

[81] Topalian SL Drake CG Pardoll DM. Immune Checkpoint Blockade: A Common Denominator Approach to Cancer Therapy. Cancer Cell (2015) 27(4):450–61. doi: 10.1016/j.ccell.2015.03.001 PMC4400238 25858804

[82] Fiorentini C Grisanti S Cosentini D Abate A Rossini E Berruti A. Molecular Drivers of Potential Immunotherapy Failure in Adrenocortical Carcinoma. J Oncol (2019) 2019:6072863. doi: 10.1155/2019/6072863 31057613 PMC6463568

[83] Grisanti S Cosentini D Laganà M Volta AD Palumbo C Massimo Tiberio GA. The Long and Winding Road to Effective Immunotherapy in Patients With Adrenocortical Carcinoma. Futur Oncol (2020) 16(36):3017–3020. doi: 10.2217/fon-2020-0686 32857613

